# Mother-to-mother therapy in India and Pakistan: adaptation and feasibility evaluation of the peer-delivered Thinking Healthy Programme

**DOI:** 10.1186/s12888-017-1244-z

**Published:** 2017-02-23

**Authors:** Najia Atif, Revathi N. Krishna, Siham Sikander, Anisha Lazarus, Anum Nisar, Ikhlaq Ahmad, Roopa Raman, Daniela C. Fuhr, Vikram Patel, Atif Rahman

**Affiliations:** 1 Human Development Research Foundation, Mandra, Gujar Khan, Pakistan; 2 Sangath Centre, H.No. 451 (168), Bhatkar Waddo, Succour, Porvorim, Goa 403521 India; 3 Centre of Global Mental Health, London School of Hygiene and Tropical Medicine, London, UK; 4 Institute of Psychology, Health and Society, University of Liverpool, Liverpool, L69 3BX UK

**Keywords:** Perinatal depression, Peers, Low and middle income countries (LMIC), Psychosocial interventions, Thinking Healthy Programme (THP)

## Abstract

**Background:**

Perinatal depression is highly prevalent in South Asia. Although effective and culturally feasible interventions exist, a key bottleneck for scaled-up delivery is lack of trained human resource. The aim of this study was to adapt an evidence-based intervention so that local women from the community (peers) could be trained to deliver it, and to test the adapted intervention for feasibility in India and Pakistan.

**Methods:**

The study was conducted in Rawalpindi, Pakistan and Goa, India. To inform the adaptation process, qualitative data was collected through 7 focus groups (four in Pakistan and three in India) and 61 in-depth interviews (India only). Following adaptation, the intervention was delivered to depressed mothers (20 in Pakistan and 24 in India) for six months through 8 peers in Pakistan and nine in India. Post intervention data was collected from depressed mothers and peers through 41 in-depth interviews (29 in Pakistan and 12 in India) and eight focus groups (one in Pakistan and seven in India). Data was analysed using Framework Analysis approach.

**Results:**

Most mothers perceived the intervention to be acceptable, useful, and viewed the peers as effective delivery-agents. The simple format using vignettes, pictures and everyday terms to describe distress made the intervention easy to understand and deliver. The peers were able to use techniques for behavioural activation with relative ease. Both the mothers and peers found that shared life-experiences and personal characteristics greatly facilitated the intervention-delivery. A minority of mothers had concerns about confidentiality and stigma related to their condition, and some peers felt the role was emotionally challenging.

**Conclusions:**

The study demonstrates the feasibility of using peers to provide interventions for perinatal depression in two South Asian settings. Peers can be a potential resource to deliver evidence-based psychosocial interventions.

**Trial registration:**

Pakistan Trial: ClinicalTrials.gov Identifier: NCT02111915 (9 April 2014), India Trial: ClinicalTrials.gov Identifier: NCT02104232 (1 April 2014).

## Background

Globally, depressive disorders are the leading contributor to the global burden of disease [1–3] and are twice as common in women compared to men, showing a particular high prevalence among women of child-bearing age, especially during pre and postnatal period [4]. In a meta-analysis of 47 studies from low and middle-income countries (LMICs), the average point prevalence of depressive episode was reported to be 15.6% during pregnancy and 19.8% in the postpartum, with substantially higher rates found in South Asia [1]. In addition to the economic and human costs of maternal depression, children of depressed mothers are at higher risk for health, developmental, and behavioral problems [5]. Studies have demonstrated strong and independent associations with pre-term birth [6, 7], low birth weight and stunting [6, 8], higher rates of diarrheal diseases [9], and early cessation of breastfeeding [10].

Psychosocial interventions are the first-line treatment of perinatal depression [11] but, in LMIC, a key barrier to the delivery of such interventions is the lack of specialist workforce [12, 13]. In such settings, “Task-Shifting” has been recommended as an implementation strategy [12, 14, 15], and several studies have shown that psychosocial interventions can be successfully delivered to mothers by community or other front-line health workers [11, 16]. However, community health workers (CHWs) in LMIC may be overburdened [17]. This is particularly the case in countries like Pakistan, where lady health workers (LHWs)–local women employed by government CHWs’ programme to work on the mother and child health agendas, are drafted to work in other priority programmes like dengue, tuberculosis and polio prevention [18, 19]. It is not surprising that mental health interventions, in spite of their public health impact, are given less priority. Therefore, it becomes imperative to explore alternative human resources for the implementation of such interventions at scale.

## Use of peers in the delivery of mental health care

The term peer has been applied in literature to persons who share common socio-demographic characteristics with the target population and/or use their own experience of overcoming an illness to assist service users [20–22]. Peers have been used for preventative and treatment interventions in chronic diseases [23] and evidence suggests that peer support can enhance positive health outcomes [22, 24, 25], reduce mental distress [25–27] and help people to deal with psychosocial aspects of chronic disease [28].

In LMICs, there is a high unmet need for perinatal mental health interventions. In an earlier study conducted in India and Pakistan, the community, including depressed women, their family members and primary health care staff expressed willingness for psychosocial interventions to be delivered by peers [29]. However, a critical challenge for peer-delivered mental health is that the intervention, while based on sound evidence-based theory, ought to be simple enough to be delivered by a lay-person, retaining the key techniques that make it effective and acceptable. The Thinking Healthy Programme (THP) is an evidence-based psychosocial intervention for perinatal depression which was adapted for peer-delivery. The intervention is described below.

## The Thinking Healthy Programme (THP)

THP was developed in Pakistan after extensive consultation with key stakeholders [30]. It employed the core principles and techniques of Cognitive Behaviour Therapy (CBT), such as building an empathetic relationship, focusing on the here and now, behaviour activation and problem solving. The programme is fully manualized and has culturally appropriate pictorial illustrations aimed at helping mothers reflect on their thinking process and encouraging family support. It was designed to be delivered via LHWs, who were provided a three day training and monthly refreshers and group supervision. Sessions were organised into five modules covering the period from the third trimester of pregnancy to one year postnatal. Each module focused on three areas–the mother’s personal health, the mother-infant relationship, and the psychosocial support of significant others. In total 16 home based sessions were offered to mothers.

To evaluate its effectiveness, a cluster randomized control trial (RCT) was conducted in rural Pakistan with a total of 900 mothers experiencing perinatal depression. The intervention more than halved the rate of perinatal depression in the intervention arm, compared to the control group. In addition to symptomatic relief, the women receiving the intervention had less disability and better overall social functioning. These effects were sustained at 12 month follow-up [31]. THP has been adopted by World Health Organisation as first-line low intensity psychosocial intervention for perinatal depression [32], and showed the strongest effect in a systematic review evaluation interventions for the delivery of perinatal mental disorders [11].

The aim of this study was to adapt the THP so that it could be delivered by peers to depressed mothers in the community in two different settings in India and Pakistan, and to test the adapted intervention for feasibility in a sample of peers and mothers in both settings.

## Methods

The project was undertaken from January 2012 to June 2014 as part of SHARE (the South Asian Hub for Advocacy, Research and Education), an NIMH-funded collaborative hub for international research on mental health, and a collaboration between the Human Development Research Foundation, Islamabad, Pakistan, the Sangath, Goa, India, the London School of Hygiene and Tropical Medicine, UK, and the University of Liverpool, UK.

### Settings and participants

The study was conducted in Potohar, a rural sub-district of Rawalpindi, located in the province of Punjab, Pakistan, and in the North District of the state of Goa, India. Potohar is one of the seven sub-districts of Rawalpindi and is located 65 km southeast of Rawalpindi city. The local language is Potohari and the economy is mainly agrarian based. However, 35% of people are engaged in non-farming jobs, such as unskilled or semi-skilled labour, government or armed forces employment. The data was collected from the two union councils of Potohar called Banda and Jatha Hathiyal with a population of approximately 10,000–15,000 each. Both union council has a Basic Health Unit (BHU), which, apart for delivering primary health care, is the local hub for the training of LHWs. Both union councils have similar gender distribution (48% male and 52% female) and educational status (40% men and 34% women–education up till 10th grade) [33].

Goa has a population of 1.46 million, with 62.17% of the population living in urban areas. The state has a high literacy rate (male 92.6%, female 82.2%) and an even gender ratio (50.7% male vs. 49.3% female). Goa is divided into North and South Goa districts; there are 3 major cities, all with a population of less than 100,000. The most widely spoken language is Konkani, followed by Marathi, Hindi and English (http://www.census2011.co.in). Participants for the study were recruited from Asilo Hospital and Goa Medical College Hospital, both situated in North Goa. The Asilo district hospital is located in the town of Mapusa, North Goa. It is the only government run district hospital in North Goa, serving as a referral point for the surrounding health centres and communities. Goa Medical College hospital is a government tertiary care hospital which is also a teaching hospital, located close to the centre of Goa and the capital, Panaji. The pilot study was conducted in the antenatal clinics of both hospitals.

The project was undertaken in two phases:

- *Phase one: Adaptation*
  The adaptation was primarily aimed at simplifying the content of the intervention manual and the delivery processes, to make the intervention more comprehensible to and deliverable by peers, who had no prior experience of delivering health care. In Pakistan, this phase consisted of conducting focus-group discussions (FGDs) with participants selected from a convenience sample of LHWs and mothers who had participated in the original THP trial [31] and agreed to take part in the discussions. The discussions aimed to explore the techniques that LHWs had found easy to learn, and were still practicing after the end of the trial, and aspects of the intervention mothers had found most useful and intuitive. In India, the adaptation of the intervention involved conducting IDIs and FGDs with stakeholders (pregnant women, family members and healthcare providers) and two theory of change workshops at the Asilo district hospital where trial recruitment was to take place. The sample was recruited by screening antenatal clinic attendees at the district hospital and interviewing them and their family members, with their informed consent. It was aimed to explore further additions to the intervention contents, who best could deliver it, and logistical issues in its delivery. The topic guides for both sites followed a semi-structured format and were pilot tested.
- *Phase two: Feasibility testing*
  The intervention, following its adaptation was field-tested for feasibility in rural Rawalpindi and in Goa. Recruitment of peers was a three stage process; the first stage involved sharing the criteria for peers’ recruitment outlined in Singla et al., [29] (see Table 1) with the LHWs in Pakistan and Anganwadi workers in India. They were well-embedded in their communities and facilitated the identification of the peers with these characteristics. Following this, at both sites, peers were interviewed by the intervention team leads and were offered a training. The final stage involved assessing their counselling skills and understanding of the intervention via role plays, during the training, using the quality assessment forms (described below). Those meeting the competency criteria were selected.

**Table 1:** Selection criteria for peers

Domain	Criteria^a^
Education/ Qualification	Pakistan: Minimum 10 years of schooling (equivalent to GCSE) India: Minimum 7 years of schooling (equivalent to 7th grade)
Experience	Similar socio-demographic background to the target population Similar life experiences to that of the target population Emotional maturity/range of life experience Motherhood experience (India: youngest child preferably at least 3 years old)
Personal attributes	Willingness to learn new skills Good interpersonal skills Ability to relate to mothers and their families Trustworthy, patient, non-judgmental, respectful and compassionate Energy/drive and enthusiasm
Knowledge	Some understanding of maternal and child health issues
Other	Fluent in local language
Requirements	Mobile, able to move in the community freely including if the target population is slightly far off from her place of residence.

^a^Criteria common for both sites unless indicated
  At both sites, women living in the catchment areas of the respective study sites, who were 18 years or older, in their second or third trimester of pregnancy or had infants up to four months of age were screened for depression using the Patient Health Questionnaire (PHQ-9). Potential participants were identified, in Pakistan through the LHWs (they maintain a record of all women, who are pregnant or have children less than one year, in their catchment areas) and in India women attending the antenatal clinics of the two hospitals. All potential participants were screened by the trained researchers, following their informed consent. All those who scored ≥ 10 on the PHQ-9 were provided with further information about the study and enrolled after obtaining informed consent. Mothers who had a physical condition requiring on-going treatment were excluded.
  Data was collected from all peers and mothers in both sites, up to six months of intervention delivery, between January 2014 and June 2014 through IDIs and FGDs. Data were collected in vernacular languages (primarily Konkani and Hindi in Goa, and Urdu in Rawalpindi) by multilingual trained researchers. In both contexts, topic guides were piloted and appropriate revisions were made. All data were recorded, either via digital audio recorders or by taking notes.

### Ethical considerations

This study followed key standards in research ethics. Informed consent was obtained from all participants before conducting interviews. Participants were interviewed at a venue of their choice. In any situation where privacy during the interview was compromised, participants were given the choice of re-scheduling the interview. During transcription of interviews, all identifiable information was anonymized and kept under lock and key. Plans were put in place to deal with any untoward effects such as a participant getting distressed during the interview or disclosing matters of serious nature. For instance, in case of discomfort or distress participants were offered the opportunity to stop or break from the interview or to speak to another member of the research team, trained in counselling skills. Peers were provided regular supervision to identify and address any work related issues.

### Data analysis

Data analysis was carried out alongside data collection using a Framework Analysis approach [34–36]. Interviews were firstly analysed manually by the respective team leaders in both sites (NA and AL/RR/RNK) and then reviewed by the other members of the team. The process involved familiarisation with the raw data through reading and re-reading the transcript and identifying main themes in the data. Key issues and recurrent themes were highlighted and the thematic framework for each set of respondents were developed by collating them into groups. Discrepancies were resolved through mutual discussions, going back to raw data and referring to field notes. Quotes from the raw data were included in the thematic framework as supporting evidence. Each quote was referenced so that it could be traced back to the original text if needed. Thematic frameworks were compared and contrasted between the two sites. Each theme and its sub-themes in the thematic framework were given an index number. These index numbers were used to code the transcripts (indexing). Charts were made for each theme across all respondents by lifting and summarising the indexed data from the transcript.

## Results

### Phase 1: adaptation

**Fig. 1:**
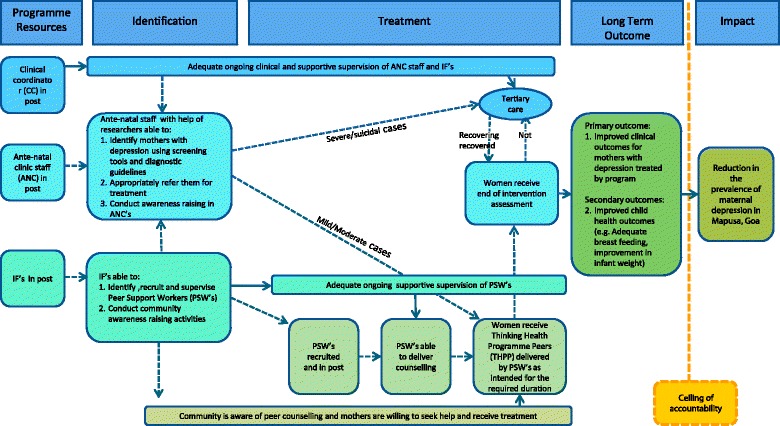
Theory of change map after formative research in India

In Rawalpindi, four FGDs were conducted at the BHU, two with LHWs (*n* = 16) previously involved in delivering THP and two with mothers (*n* = 15) who received the THP. In India, as THP was being introduced for the first time, 61 IDIs were conducted with depressed (*n* = 23) and non-depressed mothers (*n* = 16), their family members (*n* = 15), CHW (*n* = 4), specialist health care providers (*n* = 3); three FGDs were conducted, attended by 21 CHWs including multipurpose health workers and ancillary nurse midwives, and four specialist care providers (pediatricians) to evaluate its acceptability and necessary adaptation, see Table 2 below. Two workshops were conducted with hospital staff, one each at the beginning and end of the adaptation phase to develop a theory of change conceptual map for the intervention (Fig. 1 below). This identified the programme resources required, the need for adequate supervision of peers, and the intended outcomes for mothers with depression. The findings from both sites were used to inform adaptation.

**Table 2:** Number of IDIs and FGDs conducted in both settings during adaptation and feasibility testing phase

	India		Pakistan	
	IDIs	FGDs	IDIs	FGDs
Data Collection: Adaptation Phase				
Depressed mothers	23	-	-	2 (*n* = 15)
Non-depressed mothers	16	-	-	-
Family members	15	-	-	-
Community health workers	4	2 (*n* = 21)	-	2 (*n* = 16)
Specialist care providers	3	1 (*n* = 4)	-	-
Total	61	3	-	4
Data Collection: Feasibility Testing Phase				
Mothers who received the intervention	12	-	21	-
Peers		7 (*n* = 3 to 11 each focus group)	8	1 (*n* = 8)
Total	12	7	29	1

#### Adaptations to content

**Fig. 2:**
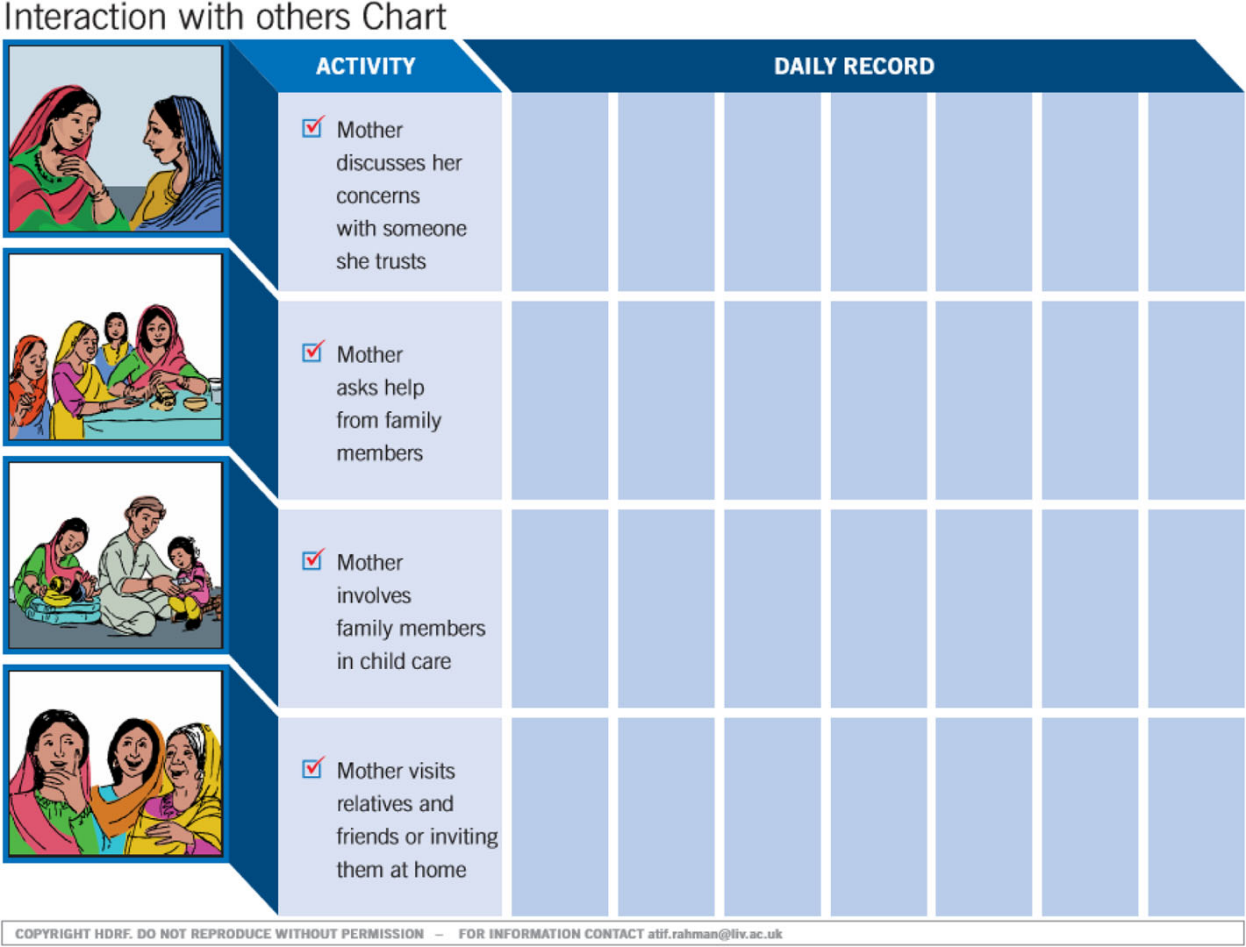
Example of a health chart. Excerpts from the Thinking Healthy Manual (peer delivered), reproduced with permission from the Human Development Research Foundation

*Emphasis on behaviour activation*: One of the steps in the original THP was identifying and modifying maladaptive thoughts using pictures and a-priori examples (simplified cognitive restructuring). Many LHWs felt this was a relatively harder skill to learn and practice. Therefore, THPP focussed more on behaviour activation and less on cognitive restructuring. Health charts with pictorial illustrations aimed to improve mother’s personal health, her social support and bonding with her infant were used to set goals and to monitor mother’s healthy activities between the sessions (see Fig. 2 below). As the intervention focussed on the perinatal period, most of the activities revolved around child-care, encouraging other family members to support these activities so the mother could derive more pleasure out of them, and be able to find some time for her own rest and self-care.

*Standardisation of health messages*: The original THP, designed for LHWs, was cross-referenced with key general health messages that LHWs routinely delivered. These health messages were standardised for peers who have no formal health background. For example, instructions on taking iron tablets during and after pregnancy, or observing exclusive breastfeeding till the age of 6 months, were added in very clear language to the training materials. Peers were advised to refer women with general health needs to LHWs in Pakistan or Anganwadi workers in India to avoid any potential clash of roles.

**Fig. 3:**
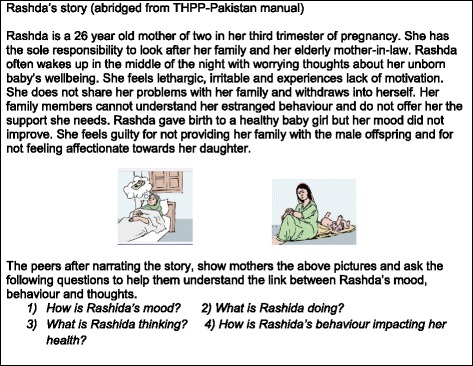
Example of a CBT-based narrative and illustrations to facilitate delivery. Excerpts from the Thinking Healthy Manual (peer delivered), reproduced with permission from the Human Development Research Foundation

*Use of illustrations and narratives:* The original THP relied heavily on illustrations with brief captions. Some of these were expanded into longer vignettes with CBT-based narratives to facilitate delivery of the sessions by peers through ‘story-telling’ (see Fig. 3) Culturally appropriate illustrations depicting different moods, behaviour and thoughts were used to encourage participants identify links between unhelpful behaviour, unhelpful thoughts and depressed mood. The stories were built further as the session progressed, with illustrations encouraging participants to identify helpful behaviours that had a favourable impact on mood. By relating with the characters in the stories and pictures, participants were able to apply the same process to their own situations.

*Simplification of structure and language*: The manual was carefully screened for any jargon, which was replaced with everyday language (Fig. 3).

#### Adaptations to delivery

*Reduction in the number of sessions*: The intervention started in the second or third trimester of pregnancy and continued until the sixth month postnatal. In India, sessions were reduced to a minimum of 6 to a maximum of 14 (according to the needs, circumstances and ability of the women to attend) and were delivered through individual sessions. In Pakistan, the sessions were reduced from 16 to a maximum of 10 individual sessions (four during prenatal period and six during postnatal). Table 3 details the period and frequency of session delivery. Additionally, four group sessions were integrated into routine monthly ‘women’s groups’ organised by LHWs for all perinatal women (i.e. including non-depressed women). Peers delivered the groups in partnership with LHWs, and included general psychoeducation and child development information. Peers motivated depressed mothers to attend the groups in order to reactivate their social networks.

**Table 3:** Period, format and frequency of session delivery for the THPP

Period of session delivery	Session numbers	Format	Frequency
In Pakistan			
Prenatal: From second or third trimester of pregnancy onward	1–4.	individual session	Varies from weekly to fortnightly to monthly depending on expected date of delivery and mother’s availability.
	1	group session	Monthly
Postnatal: Until six months post pregnancy	5–7	individual session	Fortnightly
	8–10	individual session	Monthly
	2–4	group session	Monthly
In India			
Prenatal: From second or third trimester of pregnancy onward	1–6	Individual sessions	Varies from weekly to fortnightly to monthly depending on expected date of delivery and mother’s availability.
Postnatal: Infancy (0–2 months)	7–10	Individual sessions	Fortnightly
Early Childhood (3–4 months)	11–12	Individual sessions	Monthly
Middle Childhood (5–6 months)	13–14	Individual sessions	Monthly

During the feasibility study maximum of 10 (seven individual and one group) and four (individual) sessions were delivered in Pakistan and India respectively

*Cascade training and supervision*: The paucity of specialists in LMIC, and the fact that a scaled-up version of THPP would require a large number of locally-based peers, necessitates cascade training and supervision. In THPP, a specialist master trainer (mental health specialist), trained and supervised a group of non-specialist trainers (University graduates), who in turn provided training and supervision to groups of peers. The training length was increased and followed by the field-training (training conducted on-site with actual mothers). Peers received fortnightly supervisions to ensure continuous experiential learning, explore work related stress and to maintain their motivation. Quality of the intervention was ensured through assessing peers’ competencies, on the assessment forms, during direct observations in Pakistan and audio recorded sessions in India. Peers were assessed on their competencies; to engage with the mothers using basic counselling skills (such as empathy, interpersonal and problem solving skills) and on intervention specific skills (such as identifying and practising healthy behaviours and involving families). Responses were recorded on three point Likert Scale (from 0 not done to 2 done well). A score of 80% or above indicated satisfactory level of competency. Any gaps in the knowledge and skills were addressed during supervisions.

*Use of Job-Aides*: Job-Aides with illustrations and captions summarising the narratives were developed for delivery of key messages and to facilitate the delivery of sessions in a structured fashion.

*Boundary Setting*: As opposed to the common notion of the professional counselling environment with strict boundaries, peers were allowed to have more informal boundaries i.e. through sharing their own personal experiences with the mothers, arranging sessions at the mothers’ convenience and being flexible with the time period of the session.

The same principles were applied to the adaptations of the intervention at both sites, and separate manuals were produced taking into account the contextual differences in Pakistan and India. The key features made to the THP are outlined in the Table 4 below.

**Table 4:** Key features of THP adaptation

Original THP	THPP–Pakistan	THPP–India
	Format of delivery	
LHW-delivered 16 home based individual sessions.	Peer-delivered 10 home based individual sessions & 4 group sessions.	Peer-delivered 6 to14 home based individual sessions.
Rationale: Fewer individual sessions as period of delivery was shorter for THPP. Introduction of group sessions in Pakistan based on formative research findings and evidence from the literature indicating that groups could be helpful for maternal depression.		
Training		
Training of LHWs: Three days classroom training conducted by mental health specialist.	Training of peers: Five days classroom training and field training conducted by the THPP trainers. Training of THPP trainers: 20 h classroom training and six month internship period conducted and supervised by the master trainer.	Training of peers: Five days classroom training comprising of eight sessions, delivered in two phases (the antenatal phase and the postnatal phases) and the field training conducted by the master trainer.
Rationale: In order to develop a more sustainable model, peers were trained by non-specialist THPP trainers in Pakistan. Field training was introduced in both settings to build peers’ competency and fidelity to the intervention.		
Intervention material		
Reference Manual & Health Calendar	Reference Manual, Job-Aids and Health Calendar–consisting of health charts aimed towards behavioural activation of the mothers.	Field Guides and Activity Workbook.
Rationale: The Job-Aids/Field Guides were introduced containing step-by-step instructions to facilitate peers’ in delivering of sessions. In India, pictorial illustrations were adapted and narratives were introduced to make the intervention culturally relevant to the setting.		
Supervision		
LHWs were supervised through monthly group supervisions by the mental health specialist.	Peers were supervised by the THPP trainers through regular monthly group and field supervisions. THPP trainers were supervised fortnightly by the mental health specialist.	Peers were supervised by the THPP trainers through fortnightly group supervisions and two individual supervisions during their internship period.
Rationale: Cascade model is a relatively sustainable model because it requires fewer specialist workers. In Pakistan, frequent field supervisions to ensure continuous experiential learning, quality to the intervention and to maintain peers’ motivation. In India sessions were audio recorded and discussed during supervisions in order to provide feedback to the peers and to ensure quality.		
Emphasis on behaviour activation		
More discussion during supervisions and sessions delivery on cognitions.	More emphasis during supervisions and sessions delivery on behaviour.	Emphasis on using behaviour activation strategies during delivery of sessions and as focal point of discussion during supervisions.
Rational: Based on formative research findings emphasis was given to behaviour activation. This strategy enabled the intervention to be comprehensible and deliverable by peers with no prior experience of delivering health care. To facilitate peers’ supervisions through non-mental health specialist, requiring less specialist skills.		
Payment		
LHWs were paid a regular salary.	Peers were paid only the sustenance allowance for travel to trainings and supervision.	Peers were paid a fixed amount for every session they successfully delivered.
Rational: In Pakistan, the rural context was conducive to volunteerism. In India in order to ensure peers’ continuous motivation and engagement with the programme. See Singla et al., 2014 [29].		

### Phase 2 – feasibility testing

Following adaptation, the intervention was tested in a feasibility study of 60 depressed mothers (Pakistan *n* = 24; India *n* = 36) who were recruited to receive peer-delivered intervention. In Pakistan 22 peers (Pakistan *n* = 9; India *n* = 13) were recruited to receive the training, out of which 17 peers (Pakistan *n* = 8; India *n* = 9) remained engaged with the project for its full duration. In Pakistan, 2 mother did not consent, two moved out of the area and the remaining 20 remained engaged and attended on the average 7.7 out of 10 sessions. In India, the pilot intervention was limited to 2–4 sessions. Of the 36 mothers recruited in the early stages of the feasibility phase, 4 mothers could not be later contacted and eight mothers either withdrew or dropped out. Out of remaining 24, 8 received 1 session, 11 received 2–4 sessions and five received more than 4 sessions. In India, mothers dropped out or received fewer sessions due to temporarily moving back with their birth families, at times for more than two months, to receive pre and post-natal care. In both settings, peers used engagement strategies such as family involvement and arranging visits at the convenience of the mothers.

The feasibility study was successfully conducted for the stipulated period of 6 months in both sites. Peer recruitment strategies were successfully employed without any problems, indicating that there are many women in the community who met the criteria, and were enthusiastic to take on such a role. No major untoward or unexpected incidents, such as worsening of symptoms or increase in interpersonal violence were reported in either site.

Qualitative data was collected through conducting 41 in-depth interviews (*n* = 29 Pakistan and *n* = 12 India) and eight group discussion (*n* = 1 Pakistan, *n* = 7 India), each focus group was attended by 3–11 participants (see Table 2 below). The trained researchers collected data at the venue of participants’ convenience (in Pakistan it was either participants’ home or BHU, in India it was participant’s home or the study office or a convenient place in the community). In Pakistan, all participants who were recruited for the pilot study, were approached for data collection. Twenty-one mothers (including the one who refused), were interviewed starting from 2 months into their intervention. Data from the peers was collected at different points in the pilot to understand their experiences during training, supervision and intervention delivery. In India data was collected from the peers before and after their antenatal and postnatal training sessions to identify changes needed for intervention delivery, supervision and training.

The section below describes how the peer-delivered intervention was perceived by the mothers and their families, and the perceptions of the peers themselves about their role as delivery agents for the intervention. Within this broad categorization, the themes that emerged from both sites are described below, illustrated by relevant quotes from the participants.

### Perspectives of mothers

#### Views about the peers as delivery-agents

Peers were perceived to have a good understanding of social and cultural issues of mothers. The mothers described the peers as receptive to their problems, sensitive to family dynamics, and easy to relate to.

*“A local person is better because she is aware of our cultural norms and is like us. As soon as she enters our house, she understands how the family relates to each other and is sensitive to these issues. She could tell how much time we can spare from our domestic responsibilities and she would stay there accordingly. In cities circumstances are different, people are liberated and women have no restrictions, I don’t think that they could relate to us”.* (Mother, Rawalpindi).

The peers’ experiences of pregnancy and motherhood was also important as most mothers felt comfortable discussing their issues, knowing that their peer had gone through similar life experiences.

*“She had shared her experience of motherhood with me and difficulties that she had faced when her child was small and then she asked me about my experience in pregnancy. So I feel like sharing my experience with her when she shares her experience of motherhood.”* (Mother, Goa)

*“My peer had a son and twin daughters, it was good that she was a mother. If she would not have been a mother she might have struggled to empathise with me. A woman who is a mother has gone through all stages of being pregnant, delivering, breastfeeding and raising her baby. She can explain things very well and we can openly talk”.* (Mother, Rawalpindi)

The intervention was home-based and family involvement was encouraged. Peers were largely welcomed by family members, who perceived them to be providing useful information for both mother and child.

*“No, nobody stopped her from coming, when she used to come, we all sit together, listen and respond to her. She takes time out to visit us for our betterment so we have to take time out from our housework to listen to her*”. (Mother, Rawalpindi)

*“I told the peer to come when my husband is at house… It is good to involve family members so that they also understand mother’s problem and should try to help her”. (*Mother, Goa)

#### Views about the intervention

All mothers found the intervention acceptable and relevant to their needs.

*I am learning new things every day, which are beneficial for me. She (peer) used to tell me about children, mothers, neighbours, home, and in-laws, almost about everything. She used to give me good information.* (Mother, Rawalpindi)

The majority of mothers reported some level of improvement following the intervention, including feeling more able to cope with life and family stressors and motivated to take positive action which led to an improvement in their mental health.

*“She told me not to be in tension, she also said if you are in tension then you phone me. At home no one is taking care of you then go and sit with your neighbour and take rest or call me and I will come to sit with you, I am your Sakhi (peer) I have to come. When she told me that I felt some happiness*.” (Mother, Goa)

The intervention was designed to improve mother-infant interaction. Most mothers reported spending more quality time with their children. They also began to prioritise their children’s need over domestic responsibilities and felt more motivated to breastfeed them.

*“If I am cleaning and if my daughter starts crying I leave my work and feed her first. Before I used to prioritise my house-work, but now if my daughter starts crying I leave my work and attend to her needs. I think that it is important to give time to your child”.* (Mother, Rawalpindi)

The majority of mothers reported that the manner in which the information was provided was effective and helpful. They felt that the narratives and vignettes were engaging and assisted them to understand their issues and to explore solutions. The illustrations were particularly helpful for those who could not read as they could relate to the images. Most mothers frequently revisited the pictorial material and succinct messages left behind by peers, which served as a reminders and motivator to engage in useful activities between sessions.

*“At times I used to feel angry, but then I think about the mother in the pictures, who is affectionate towards her child and I feel I should do the same. When I feed my daughter she tries to grab my fingers, then the image of mother and child interacting with each other come to my mind and makes me think that I should not be angry with her as she is just a baby”.* (Mother, Rawalpindi)

#### Views on potential untoward effects of the intervention

In Pakistan while most mothers preferred peers to be from the same locality, a few were concerned about confidentiality.

*“It might be difficult for me to talk about my problems as she lives in my village and I see her every day. I am worried she will talk to others and if my mother-in-law will hear anything from anyone I will be in trouble”.* (Mother, Rawalpindi)

A related concern was the stigma of a mental condition. Even though the intervention was designed so that normal everyday terms like ‘tension’ or ‘sadness’ were used instead of medicalised terms such as ‘depression’ or ‘illness’, a minority of women were still concerned about psychosocial nature of the intervention. Only one woman discontinued intervention because of such concerns.

*“I wanted to take part in this programme, but when baji (peer) came, she said to me that your assessment has indicated that you have mental tension. I got angry, I am not mad. My in-laws didn’t like it either, they got concerned that she might spread the rumour in the village”.* (Mother, Rawalpindi)

### Perspectives of peers

#### Views about their role

In both sites, there was an enthusiastic response from potential peers to the recruitment. On interview, they continued to show enthusiasm for and satisfaction from their role, and ascribed a number of reasons for this. Some were motivated to help others for altruistic reasons.

*“Right from the first meeting, she shared her tension with me. We are providing the support that we didn’t receive when we were pregnant. Through us someone would be tension free someway like this.”* (Peer, Goa)

For other peers, it was seen as an opportunity to learn new skills and enhance their chances of more substantive employment in the future.

*“In our village there are limited opportunities for women to gain knowledge and learn new skills. My experience of working as a peer will hopefully improve my chance to gain a job as a LHW”.* (Peer, Rawalpindi)

Equally important for the peers’ motivation and job-satisfaction was acceptance and appreciation of their role by the mothers’ families and of the communities they served. Acceptance, and in many cases appreciation, gave them an enhanced social status. The majority of the peers did not experience any resistance and have reported approval of their role by their communities.

*“Our programme is such that families agree to it and like it. They know that our team is giving them the right information. They recognise that their children are going to benefit from it too. They are also passing on the information to their relatives”.* (Peer, Rawalpindi)

#### Views about the intervention

The content of the intervention was perceived to be simple and acceptable. The key messages given were relevant to the mothers’ current circumstances and intuitive to both its receiver and deliverer. They were targeted to improve mothers’ wellbeing and were delivered in a sensitive and culturally appropriate manner.

*“I liked my Sakhi (peer) because she was asking me to take care of myself. When there was nobody, I did not take care of myself, but when she started working with me I have started looking after myself, realising I have to do so for my child.”* (Mother, Goa)

The intervention uses the power of imagery and narratives to encourage mothers to become more active. Similar to the views expressed by mothers, the majority of peers felt that both the stories and pictures were effective tools for communicating, that mothers could relate to them and that they were motivational in improving mothers’ wellbeing.

*“Mothers who cannot read understand the information through looking at these pictures in the manual. They talk about the pictures but actually they relate these to their own problems”.* (Peer, Rawalpindi)

During each session the peer assessed the mother’s mood by recording her response on a mood chart (pictorial illustrations representing mood on a 5-point scale ranging from very sad to very happy). In addition, they relied on accounts given by the mothers and their family members about their general wellbeing. Almost all peers received positive feedback from mothers or their families about the intervention.

*“You can tell that a mother is feeling better through her response to the mood chart and when she smiles and when she shares her thoughts and feelings with you.”* (Peer, Goa)

#### Views about training and supervision

The majority of the peers were satisfied about the adequacy of their training and supervision. They felt it increased their knowledge and adequately prepared them to face potentially challenging situations during their work. The peers found the concepts taught intuitive, and could relate them to their own experiences.

*“Training was good, we learnt a lot from this training, such as how to take care of one’s self, how to overcome stress related problems and the importance of sharing one’s problems. The training helped us to help others.”* (Peer, Goa)

In Pakistan, the peers received alternate fortnightly group supervisions and field supervisions conducted by their THPP trainers. In India, monthly supervision involved peer assessment of the audio recorded session using a Therapy Quality Scale specifically developed for this purpose. Most peers in both sites felt that the supervisory support was adequate and supervisors managed to deal with their issues effectively.

*“Group supervision is helpful to discuss and understand issues through listening to the audio recording. We share our experiences and challenges during supervision and get more idea from other colleagues and supervisors to help the mother. If we have missed something during the session, we learn about it when we listen to the audio recording. So, we can improve ourselves”* (Peer, Goa)

#### Views on potential untoward effects of the intervention

In Pakistan, a peer experienced stress because a mother wanted to withdraw from receiving the intervention. Her concerns were addressed sensitively during supervision.

*“I was awake all night because a mother told me not to come anymore. I got tensed, I didn’t speak to anyone, I was thinking I might have upset her or she didn’t like me, why she didn’t like me, why did she behave like this…what was her reason for doing that”* (Peer, Rawalpindi).

#### Views on motivation of the peers

In Pakistan, peers were happy to work on a voluntary basis, however, in Goa, India, the peers expressed the need for some kind of incentive structure for this programme to be successful and to hold their interest.

*“People ask us why we are going for this job if it does not pay us. If we got monthly salary, then, it would be good. We will also be interested in working.”* (Peer, India).

## Discussion

In India and Pakistan where rates of perinatal depression are high and the treatment gap over ninety percent, a CBT based intervention (THP) was adapted for delivery by peers, and tested for feasibility. Key adaptations included a greater emphasis on behavioral activation rather than cognitive aspects of the intervention; using narratives and pictures to gently challenge unhelpful thinking and behaviour, and encourage helpful ones; and use of simple everyday language that both the peers and the mothers could relate to. Brief class-room training was supplemented with regular group and field-supervision by non-mental health specialists, who in turn were supervised by a specialist therapist (cascade model of training and supervision). In a feasibility study of the adapted intervention, most women who received the intervention remained engaged as they perceived it to be useful, and viewed the peers as effective delivery-agents. The simple format using narratives, pictures and everyday terms to describe distress, and an emphasis on behavioural activation, made the intervention relatively easy to understand and deliver. Peers found most of the concepts intuitive and easy to convey to the mothers, and both the mothers and peers found family involvement, shared life-experiences and personal characteristics greatly facilitated the intervention-delivery. A minority of mothers had concerns about confidentiality and stigma related to their condition, and some peers felt the role was emotionally challenging. These concerns would require to be addressed in training and continued supervision. Even though peers found it difficult to navigate outside world initially (being primarily house-makers), they were able to do it successfully and towards the end of the pilot, they identified themselves as peers in this programme as much as they were home-makers.

The adaptation and feasibility phases identified a number of challenges, some of which were site-specific, that needed to be addressed before the intervention could be tested for effectiveness. These included the need for monetary incentives for peers in Goa, the cultural custom of returning to one’s mother’s house for child birth, and the migrant population attending the district hospital in Goa, scheduling sessions while the mother seemed to not prioritise her sessions with the peer. Furthermore, the peers found it hard to use the field guides (the manual) though they were largely pictorial. Peers also found it challenging to prioritise supervision at times. Some of these challenges were addressed iteratively in a second phase of the feasibility testing, before the trials began, while the remaining challenges were addressed through the methodologies used in the trial (e.g. excluding mothers who would not remain in the trial areas, use of telephone sessions, having better illustrations with less text). In Pakistan, the setting was more rural and the elevated status of peers and goodwill of their community was seen as a sufficient reward. Communities were more closely-knit, so confidentiality of any sensitive information was emphasized, and peers encouraged to divert focus to the child and mothers’ well-being if they found themselves being involved in interfamilial conflict.

In the absence of any specialist mental health services, non-specialist led services are likely to be the only alternative in low-income settings [12, 14, 15]. This study adds to the small but growing literature on the use of peers for delivery of maternal mental health in LMIC [37–39].

For a psychological intervention to be acceptable, it has to be culturally appropriate, contextually relevant and usefulness to the target population [25, 40]. The original THP (delivered through LHWs) took into account the individual and sociocultural context of the patient’s problems and produced significant positive outcomes [31]. The same intervention, with adaptations, when delivered by peers with no prior experience of health-care delivery, was found to be culturally acceptable and relevant to the mothers’ needs. There are examples of other peer-delivered interventions in low-income countries that were successful because of being contextually relevant to their recipients [37, 39, 41], and others that failed because this critical element was not addressed [42, 43]. Peers seem to be especially suited to delivering the intervention in a culturally and contextually appropriate fashion, as they have shared life experiences and sociodemographic characteristics.

The adapted THP makes use of stories, peers’ own experiences and illustrations to facilitate delivery. The use of stories, metaphors and analogies in CBT, when assessing suitability for treatment, challenging unhelpful styles of thinking, and addressing maintaining behaviours, has been advocated [44]. The collaborative development of stories can enhance rapport, enable clients to gain a new perspective upon their problems, increase personal impact and clarity of meaning, and reinforce clients’ motivation to effect therapeutic change. This was found to be a particularly beneficial strategy for delivery by peers in South Asian settings, which have a strong narrative tradition.

Adequate training and supervisory support is essential for any programme. A meta-synthesis found that effective training and supervision were vital for the success of the peer support role [45]. In the current study, supervision sessions provided experiential learning and ample emotional and practical support, which kept them motivated and helped them to deal with the challenges in the field. However, the findings indicate that work is likely to be emotionally challenging for peers who have limited exposure to mental health training, and therefore supervision assumes added importance. These study findings are consistent with the evidence from other studies with lay workers. For example, a study conducted in Nepal showed that increasing training and supervision sessions was effective in sustaining peers’ interest and motivation [46]. In India, training and supervision was helpful in increasing peers’ credibility in the community [47], in Uganda it provided continuous motivation [48], and in South Africa it helped them dealing with challenging issues [49].

Future qualitative studies of peer-delivered programmes should consider exploring the long-term impact of peer volunteering. Knowledge of factors, which could impact peers’ engagement in programmes over time such as level of motivation, supervisory support and job stress, could help to plan long term peer-delivered programmes. Another area of research could be exploring how peers have progressed over time in terms of their personal development, autonomy and career progression, and how this might contribute to the women development agenda. Future research should also consider exploring the viewpoint of policy makers, programme managers and community leaders. This could give a better understanding of factors that could either facilitate or hinder the establishment and management of peer-delivered health programmes–leading to better planning. Finally, such peer-delivered programmes should be subjected to more rigorous evaluation–the current programme is being evaluated through two randomised controlled trials in Rawalpindi and Goa.

### Limitations

A major limitation was that it was a short-term study covering a period of six months in which the peers were deployed in this role. Therefore the long-term feasibility of the approach could not be explored and some significant questions remained unanswered. A key question was the motivation of peers to work in the longer-term in a voluntary or semi-voluntary capacity. Although this was a short timeframe, it provided enough input to be able to move towards testing the intervention for effectiveness. The RCTs from both sites will provide more information on the effectiveness of THPP, as well as the barriers and facilitators of delivery by peers. In India, it was clear even in this short time frame that volunteering was not a feasible option, while in Pakistan, it appeared to be feasible. Our previous work [29] shows that volunteering is context-specific and more acceptable in Rawalpindi, a closer-knit rural agrarian society than Goa, a more urban and transitional society. Other aspects include the emotional impact (such as burnout) of working with depressed mothers over an extended period; their longer-term relationship with the families, and; long-term collaboration with government health providers. These issues have implications for future research.

Other limitations include use of convenience sampling during the adaptation phase and social desirability factor. Social desirability on behalf of the participants might have contributed toward positive feedback received. Furthermore, peers’ own aspirations for continued community work could have resulted in them painting a more positive picture of their experiences. The findings need to be interpreted in the light of these considerable limitations. Furthermore, the study was conducted in the South Asian culture and context, and the findings need to be generalised with caution to other settings.

## Conclusions

The current study successfully used peers to provide help to mothers experiencing perinatal depression in two South Asian settings. These findings add to the growing body of knowledge that peers can be used as a substantial resource to narrow the treatment gap for not just perinatal depression but other mental health conditions.

## Abbreviations

BHU: Basic Health Unit
CBT: Cognitive Behaviour Therapy
CHW: Community Health Worker
FGD: Focus Group Discussion
IDI: In-depth interview
LHW: Lady Health Worker
LMIC: Low and middle income countries
THP: Thinking Healthy Programme
THPP: Thinking Healthy Programme Peer Delivered
